# Decreased circulating levels of ANGPTL8 in Graves’ disease patients

**DOI:** 10.1007/s42000-019-00095-8

**Published:** 2019-03-21

**Authors:** Haoxiang Li, Mengjiao Xu, Li Zhao, Hong Xia, Yanyan Li, Xiafei Hong, Xia Deng, Jing Yuan, Yi Ding, Chang Guo, Ruirong Pan, Dong Wang, Jifang Wang, Wei Yin, Ling Yang, Guoyue Yuan

**Affiliations:** Department of Endocrinology, Affiliated Hospital of Jiangsu University, 438, Jiefang Road, Zhenjiang, 212001 Jiangsu China

**Keywords:** ANGPTL8, Graves’ disease

## Abstract

**Background:**

Angiopoietin-like protein 8 (ANGPTL8), a newly identified hormone, has been recently characterized as a metabolic regulator which can affect energy homeostasis and has interesting potentials as a metabolic disease therapy. However, little is as yet known as to whether circulating ANGPTL8 levels are altered in thyroid dysfunction. This study measured serum ANGPTL8 levels in patients with Graves’ disease and explored the correlations between its serum levels and thyroid index in Graves’ disease.

**Methods:**

The concentration of ANGPTL8 was analyzed in blood samples of 128 well-characterized individuals whose anthropometric parameters, biochemical parameters, and thyroid index were measured. The participants were divided into Graves’ disease patients (*n* = 60) and healthy control subjects (*n* = 68). Logistic regression was used to evaluate the relationship between ANGPTL8 and Graves’ disease.

**Results:**

Serum ANGPTL8 levels were more significantly decreased in Graves’ disease patients than in healthy control subjects (177.67 ± 135.07 vs 326.41 ± 194.72 pg/mL; *p* < 0.001). Serum ANGPTL8 was negatively correlated with free triiodothyronine (FT3), free thyroxine (FT4), and thyroid peroxidase antibodies (TPOAb) while being positively correlated with thyrotropin (TSH). Logistic regression analyses demonstrated that serum ANGPTL8 was significantly associated with Graves’ disease (*p* < 0.05).

**Conclusions:**

Circulating concentrations of ANGPTL8 showed a significant reduction in Graves’ disease patients. Thus, it is suggested that thyroid function should be taken into consideration when evaluating the results of ANGPTL8.

## Introduction

Angiopoietin-like protein 8 (ANGPTL8), which is also known as TD26 [1], refeeding-induced fat and liver protein (RIFL) [2], lipasin [3], and betatrophin [4], is an atypical member of the ANGPTL family [5]. It is a newly identified protein, composed of 198 amino acids, which is predominantly expressed in liver in humans, while mouse ANGPTL8 is highly enriched in adipose tissue and liver. Over the past few decades, increasing numbers of researchers have focused on the study of ANGPTL8. Recent studies have revealed that ANGPTL8 plays a significant role in several metabolic diseases, in particular, type 2 diabetes mellitus (T2DM) [6–8], obesity [9–11], non-alcoholic fatty liver disease (NAFLD) [12], and polycystic ovary syndrome (PCOS) [13].

Hyperthyroidism is a pathological disorder in which excess thyroid hormone is synthesized and secreted by the thyroid gland. Prevalence of hyperthyroidism is 0.8% in Europe [14] and 1.3% in the USA [15]. The most common cause of hyperthyroidism is Graves’ disease, followed by toxic nodular goiter. Graves’ disease is an autoimmune thyroid disease with such symptoms as weight loss, heat intolerance, tachycardia, and mental disturbances. Recently, many studies have demonstrated that changes in thyroid function are associated with changes in energy metabolism [16–18]. Graves’ disease has deleterious metabolic effects on lipid, carbohydrate, and amino acids metabolism [19, 20].

Previous research has shown that the levels of circulating hepatocyte- and adipocyte-derived metabolic regulators were altered in patients with thyroid diseases, such as leptin, resistin, adiponectin, and FGF21 [21, 22]. Recently, Han et al. reported that circulating ANGPTL8 was elevated in patients with overt hypothyroidism and subclinical hypothyroidism [23]. However, little is to date known as to whether circulating ANGPTL8 levels are altered in Graves’ disease. In this study, we aimed to determine the levels of serum ANGPTL8 in normal control subjects and patients with Graves’ disease while further exploring the factors associated with ANGPTL8 levels.

## Materials and methods

### Subjects

A total of 128 subjects were recruited in this study: 68 healthy control subjects and 60 patients diagnosed with Graves’ disease who were prepared to receive ^131^I treatment. GD patients were required to discontinue antithyroid drug (ATD) therapy for more than 2 weeks before the treatment. Pretreatment protocol also included obtainment of informed consent, negative pregnancy test, ultrasound measured thyroid or nodule volume, hormonal status, and thyroid scintigraphy. All patients were placed on a low-dose iodine diet 10 days prior to therapy. Individuals with diabetes mellitus, renal disease, nephritic range proteinuria, coronary heart disease, heart failure, peripheral artery disease, cerebrovascular event and malignancy, or other known major diseases were precluded from the study.

### Diagnostic criteria

The diagnosis of Graves’ disease was documented by clinical and biochemical evidence of hyperthyroidism, diffused goiter, and the presence of positive thyrotropin (TSH) receptor autoantibody tests, diffusely increased ^131^I (iodine-131) uptake in the thyroid gland, or the presence of exophthalmos.

### Anthropometric and biochemical measurements

Height and weight of all patients were measured to the nearest 0.1 cm and 0.1 kg, respectively, and BMI was calculated as weight divided by height squared (kg/m^2^). Blood samples were withdrawn from an antecubital vein after a 10-h overnight fasting. After clotting, blood specimens were separated by centrifugation. Serum samples were subsequently stored at − 80 °C until immediate analysis of ANGPTL8. Serum glucose levels were determined using the glucose oxidase method. Alanine aminotransferase (ALT), aspartate aminotransferase (AST), gamma glutamyl transpeptidase (GGT), low-density lipoprotein cholesterol (LDL-C), high-density lipoprotein cholesterol (HDL-C), total cholesterol (TC), apolipoprotein A (APOA), apolipoprotein B (APOB), and triglycerides were measured by the kinetic method (Beckman Coulter Inc., Brea, CA, USA). Serum free thyroxine (FT4), free triiodothyronine (FT3), thyrotropin (TSH), thyroid peroxidase antibody (TPOAb), thyroglobulin (Tg), antithyroglobulin antibody (TgAb), and TSH receptor autoantibody (TRAb) were tested with a chemiluminescence immunoassay (Beckman Coulter Inc., Brea, CA, USA). The radio iodine uptake (RAIU) value was obtained 2, 6, and 24 h after an oral tracer dose (about 1850 kBq) of ^131^I through a nuclear multifunctional instrument (MN-6110XTThyroid function instrument). Thyroidal ^131^I uptake was calculated according to the following equation: RAIU (%) = (neck counts − background counts) × 100/ (standard counts – background counts).

### Measurement of ANGPTL8

Serum levels of ANGPTL8 were determined using a commercially available human ELISA Kit (catalog no. E11644h; Wuhan Eiaab Science, Wuhan, China) with an intra-assay coefficient of variation (CV) of ≤ 4.8% and an inter-assay CV of ≤ 7.2%. A calibration curve was constructed by plotting the absorbance values at 450 nm versus the ANGPTL8 concentrations of the calibrators, by which concentrations of samples were determined.

### Statistical analysis

All statistical analyses were performed using SPSS version 16.0 (SPSS Inc., Chicago, IL, USA). Data were summarized as means ± standard deviations for normally distributed variables, medians plus percentiles (25th; 75th) for non-normally distributed variables, and frequencies for categorical variables. For comparisons between case and control subjects, the independent Student’s *t* test was used for normally distributed variables, while the non-parametric test was used for non-normally distributed variables. Categorical variables were examined by the *x*^2^ test. Partial correlation coefficients were calculated to evaluate the associations between serum ANGPTL8 and clinical and laboratory measurements. Partial correlation was used to determine the associations after adjusting for the effects of age, sex, and BMI. Binary logistic regression analyses were performed on the association of ANGPTL8, which were stratified in quartiles to estimate the odds ratio of Graves’ disease in each quartile, using the lowest quartile as the reference category. All calculated *p* values were two-sided and *p* values < 0.05 were considered statistically significant.

## Results

### Clinical and biochemical characteristics between the two groups

The clinical and biochemical characteristics of the subgroups studied (control and Graves’ disease) are summarized in Table 1. There were no statistically significant differences between Graves’ disease patients and control subjects with respect to age, sex, BMI, SBP, DBP, FPG, and APOA. Compared with those in the control group, pulse pressure, GGT, ALT, AST, triglycerides, FT3, FT4,TgAb, TPOAb, and Tg in the Graves’ disease group were significantly increased (*p* < 0.01 or *p* < 0.05), while TC, HDL-C, LDL-C, APOB, and TSH were significantly decreased (*p* < 0.01 or *p* < 0.05). The average of RAIU value and TRAb is above the normal range in the Graves’ disease group.

**Table 1:** Clinical and biochemical characteristics

	Control	Graves’ disease	*p*
Age (year)^a^	44.43 ± 13.39	41.97 ± 13.1	0.297
Sex (F/M)^b^	68(36/32)	60(41/19)	0.076
BMI (kg/m2)^a^	21.88(19.93–23.1)	21.03(19.72–22.58)	0.115
SBP (mmHg)^a^	120.32 ± 13.83	123.06 ± 12.19	0.265
DBP (mmHg)^a^	75.53 ± 10.83	72.48 ± 7.88	0.090
Pulse pressure (mmHg)^a^	44.79 ± 10.03	50.57 ± 10.84	0.003*
FPG (mmol/L)^a^	5.11 ± 0.41	5.12 ± 0.44	0.987
GGT (U/L)^a^	16.3(13–22.78)	25.15(17.6–40.78)	< 0.001*
ALT (U/L)^a^	12.65(10–21.23)	28.55(19.68–45.93)	< 0.001*
AST (U/L)^a^	1.17(1.08–1.23)	19.3(13.7–29.1)	< 0.001*
Triglyceride (mmol/L)	0.96 ± 0.28	1.13 ± 0.35	0.004*
TC (mmol/L)^a^	4.35 ± 0.44	3.35 ± 0.62	< 0.001*
HDL-C (mmol/L)^a^	1.37 ± 0.26	1.11 ± 0.25	< 0.001*
LDL-C (mmol/L)^a^	2.24 ± 0.33	1.58 ± 0.47	< 0.001*
APOA (g/L)^a^	1.11(1.02–1.17)	1.11(1–1.24)	0.921
APOB (g/L)^c^	0.88(0.78–0.93)	0.68(0.58–0.78)	< 0.001*
FT3 (pmol/L)^c^	5.2(4.29–5.67)	18.25(11.22–27.51)	< 0.001*
FT4 (pmol/L)^c^	12.56(10.96–16.37)	49.2(34.94–65.52)	< 0.001*
TSH (mIU/L)^c^	1.86 ± 1.09	0.02 ± 0.04	< 0.001*
TgAb (IU/mL)^c^	0.35(0.1–11.16)	4.85(0.73–69.83)	< 0.001*
TPOAb (IU/mL)^c^	1.36(0.83–2.48)	278.8(67.53–821.22)	< 0.001*
Tg (ng/mL)^c^	8.35(4.6–12.49)	18.35(2.13–75.05)	0.007*
TRAb (IU/mL)^a^	–	26.05(16.9–65.5)	–
RAIU (%)			
2h^a^	–	46.03 ± 1.96	–
6h^a^	–	67.65(54.53–76.18)	–
24h^a^	–	72.45(64.05–81.65)	–
ANGPTL8 (pg/mL)^a^	326.41 ± 194.72	177.67 ± 135.07	< 0.001*

Data are presented as means ± SD, *n* (%), and median (25th and 75th percentiles). A *p* value of < 0.05 was considered significant (*)

*F* female, *M* male, *BMI* body mass index, *SBP* systolic blood pressure, *DBP* diastolic blood pressure, *FPG* fasting plasma glucose, *GGT* gamma glutamyl transpeptidase, *ALT* alanine aminotransferase, *AST* aspartate aminotransferase, *TC* total cholesterol, *HDL-C* high-density lipoprotein cholesterol, *LDL-C* low-density lipoprotein cholesterol, *APOA* apolipoprotein A, *APOB* apolipoprotein B, *FT3* free triiodothyronine, *T4* free thyroxine, *TSH* thyrotropin, *TgAb* anti-thyroglobulin antibody, *TPOAb* thyroid peroxidase antibody, *Tg* thyroglobulin, *TRAb* TSH receptor

^a^Independent Student’s *t* test was used

^b^*χ*^2^ test was used

^c^Non-parametric test was used

### Serum ANGPTL8 levels

**Fig. 1:**
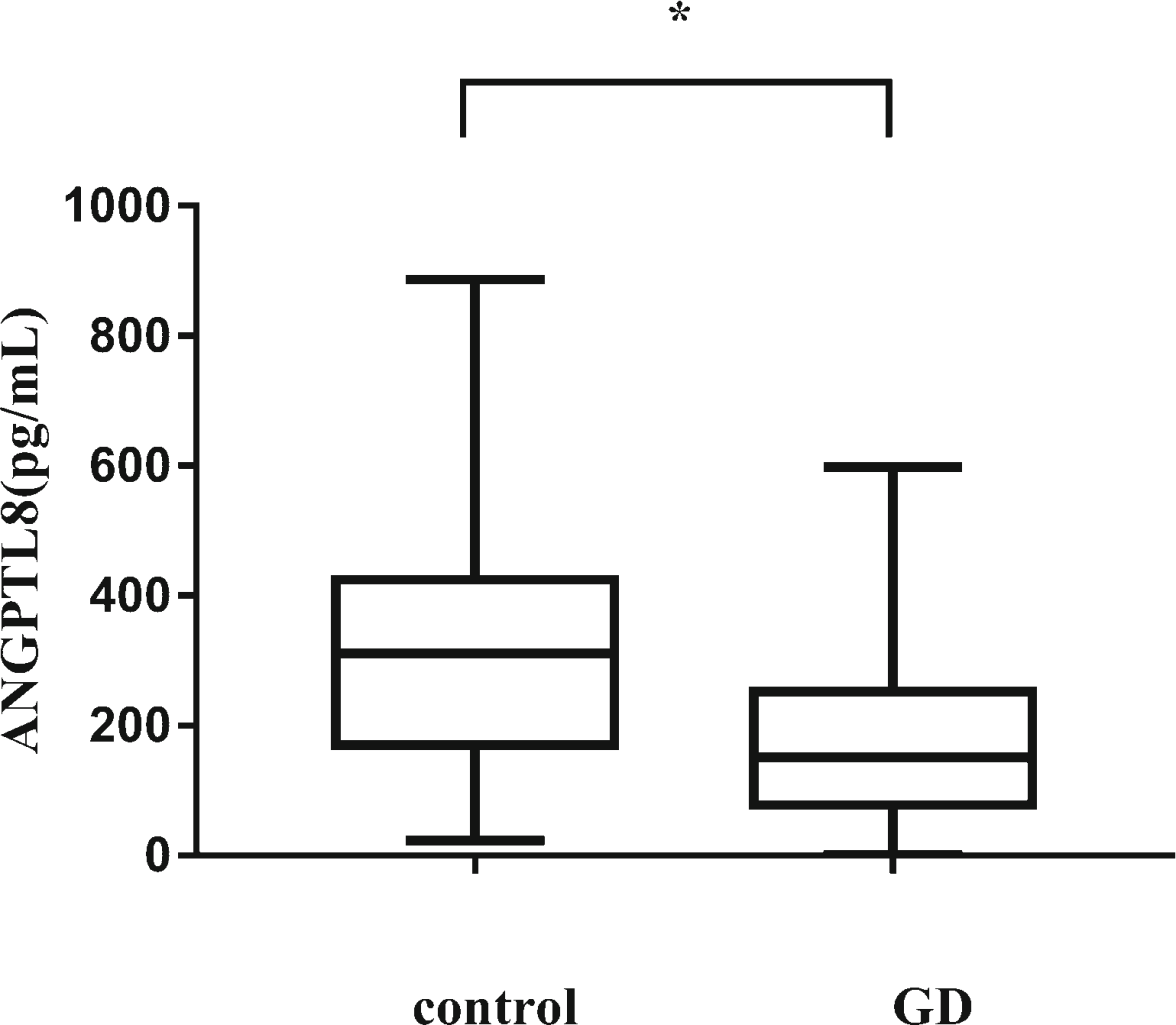
Serum ANGPTL8 was significantly decreased in the Graves’ disease (GD) group compared with the control group. *Statistically significant difference of ANGPTL8 levels between the two groups

Serum ANGPTL8 levels between the two groups were 326.41 ± 194.72 pg/mL for the control group as opposed to 177.67 ± 135.07 pg/mL for the Graves’ disease group. Compared with those in the control subjects, the levels of ANGPTL8 in Graves’ disease patients were significantly decreased (*p* < 0.001) (Fig. 1).

### The prevalence of Graves’ disease in the study subjects according to the quartiles of ANGPTL8

**Fig. 2:**
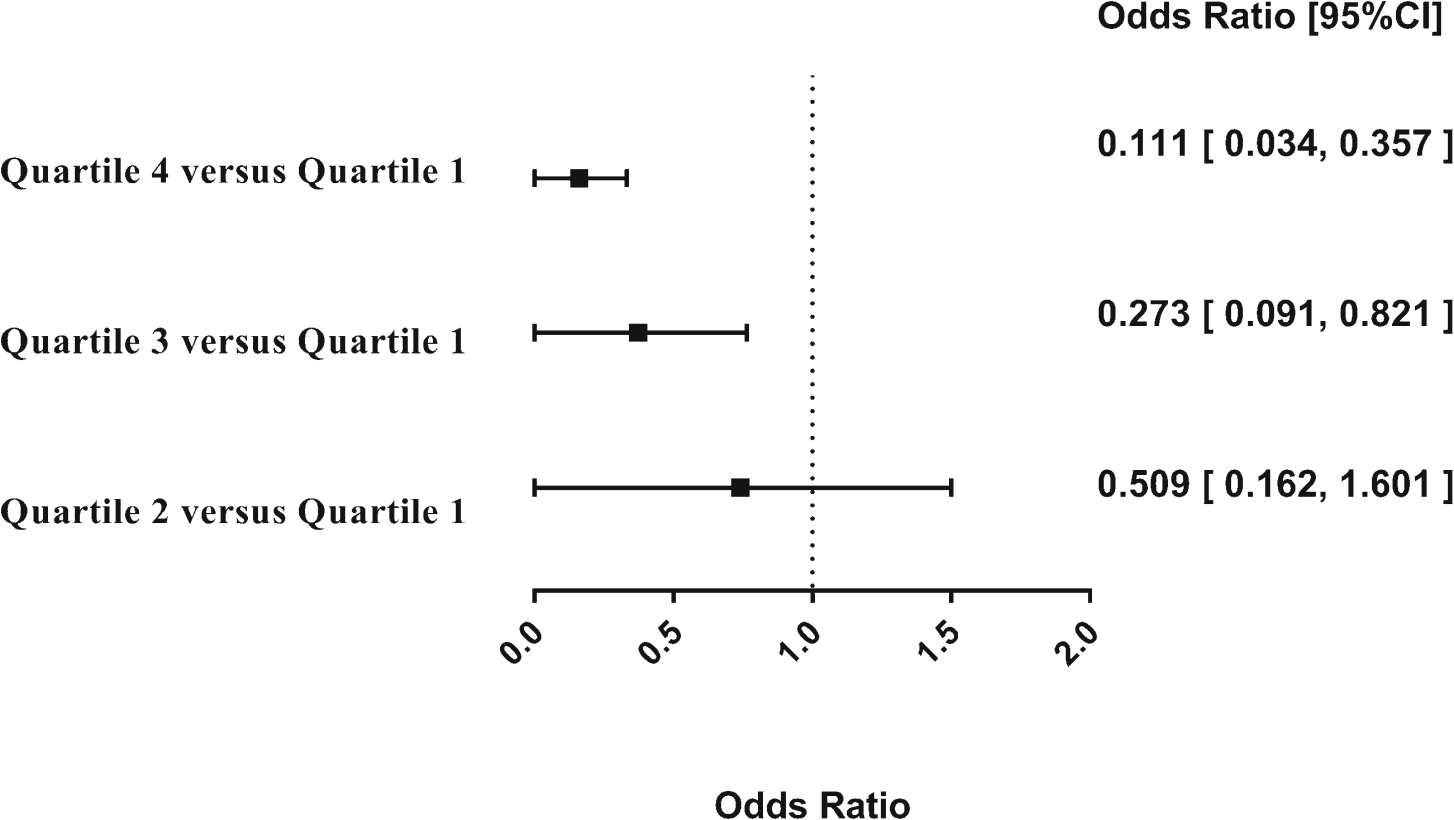
Forest plot illustrating the association of ANGPTL8 with Graves’ disease and providing data on ANGPTL8 divided into quartiles. ORs and 95% CLs are shown, and all the data are age-, sex-, and BMI-adjusted

When ANGPTL8 concentrations were stratified in quartiles (Q1, 86.16 ± 33.24 pg/mL; Q2, 184.72 ± 29.29 pg/mL; Q3, 315.59 ± 43.87 pg/mL; Q4, 564.73 ± 147.12 pg/mL), the association was most pronounced for the fourth quartile, with an OR of 0.111 (95% CI 0.034–0.357, *p* < 0.001) in the age-, BMI-, and sex-adjusted model. This association was already present but less pronounced in the third quartile (age-, BMI-, and sex-adjusted: OR = 0.273 [95% CI 0.091–0.821], *p* = 0.021) (Fig. 2).

### Relationships between serum ANGPTL8 levels and biochemical variables

Table 2 depicts a correlation analysis between the biochemical variables and ANGPTL8 after adjustment for age, sex, and BMI. ANGPTL8 was negatively correlated with FT3 (*r* = − 0.302, *p* = 0.001), FT4 (*r* = − 0.386, *p* < 0.001), and TPOAb (*r* = − 0.304, *p* < 0.001), but positively correlated with LDL-C (*r* = 0.302, *p* = 0.006), TC (*r* = 0.267, *p* = 0.006), APOB (*r* = 0.246, *p* = 0.011), and TSH (*r* = 0.324, *p* < 0.001). It was also shown that ANGPTL8 had no correlation with SBP, DBP, pulse pressure, FPG, triglycerides, HDL-C, TgAb, Tg, TRAb, and RAIU.

**Table 2:** Partial correlations analysis of variables associated with circulating ANGPTL8 levels in study subjects

	ANGPTL8		ANGPTL8(age, sex, and BMI adjusted)	
	*r*	*p*	*r*	*p*
Age^a^	0.375	< 0.001*		
BMI^a^	0.184	0.053		
SBP^a^	0.203	0.029*	− 0.01	0.920
DBP^a^	0.185	0.046*	0.02	0.843
Pulse pressure^a^	0.081	0.390	− 0.028	0.776
FPG^a^	0.109	0.222	− 0.044	0.647
GGT^a^	− 0.046	0.603	0.003	0.979
ALT^a^	− 0.119	0.182	− 0.106	0.272
AST^a^	− 0.056	0.533	− 0.069	0.476
Triglyceride^a^	0.095	0.290	0.014	0.888
TC^a^	0.333	< 0.001*	0.267	0.006*
HDL-C^a^	0.064	0.478	0.177	0.069
LDL-C^a^	0.32	0.001*	0.302	0.006*
APOA^a^	0.05	0.578	0.117	0.232
APOB^b^	0.451	< 0.001*	0.246	0.011*
FT3^b^	− 0.323	0.001*	− 0.198	0.039*
FT4^b^	− 0.386	< 0.001*	− 0.281	0.003*
TSH^b^	0.324	< 0.001*	0.195	0.043*
TgAb^b^	− 0.196	0.027*	− 0.095	0.325
TPOAb^b^	− 0.304	< 0.001*	− 0.282	0.003*
Tg^b^	0.085	0.342	− 0.127	0.189

A *p* value of < 0.05 was considered significant (*)

*BMI* body mass index, *SBP* systolic blood pressure, *DBP* diastolic blood pressure, *FPG* fasting plasma glucose, *GGT* gamma glutamyl transpeptidase, *ALT* alanine aminotransferase, *AST* aspartate aminotransferase, *TC* total cholesterol, *HDL-C* high-density lipoprotein cholesterol, *LDL-C* low-density lipoprotein cholesterol, *APOA* apolipoprotein A, *APOB* apolipoprotein B, *FT3* free triiodothyronine, *FT4* free thyroxine, *TSH* thyrotropin, *TgAb* anti-thyroglobulin antibody, *TPOAb* thyroid peroxidase antibody, *Tg* thyroglobulin, *TRAb* TSH receptor autoantibody

^a^Pearson’s correlation analysis was used

^b^Spearman’s correlation analysis was used

## Discussion

In this study, we have demonstrated for the first time, to our knowledge, that serum ANGPTL8 concentrations were significantly decreased in Graves’ disease patients compared with the control subjects. Our data showed that ANGPTL8 was negatively correlated with FT3, FT4, and TPOAb, but positively correlated with TSH. Furthermore, the trend in the presence of Graves’ disease was more apparent in the lower serum ANGPTL8 quartile group. Recently, Han et al. reported that ANGPTL8 was increased in patients with overt and subclinical hypothyroidism. In that study, a total of 122 subjects were recruited, including patients with overt hypothyroidism (*n* = 31), subclinical hypothyroidism (*n* = 30), isolated TPOAb positivity (*n* = 30), and healthy controls (*n* = 31), according to thyroid function. Furthermore, they observed that ANGPTL8 was negatively correlated with FT4 (*r* = − 0.212, *p* = 0.02), and FT3 (*r* = − 0.225, *p* = 0.01), but positively correlated with TSH (*r* = 0.438, *p* < 0.001) [23]. Tseng et al. evaluated gene expression changes in T3-stimulated cells by DNA microarray analysis with the aim of identifying the genes in liver cells regulated by T3. In that study, however, they observed that the stimulation of FT3 led to upregulation of both mRNA and protein levels of ANGPTL8, which was dependent on the thyroid hormone receptor that binds to the ANGPTL8 upstream element [24]. Further studies are needed to elucidate the mechanisms underlying decreased ANGPTL8 levels in Graves’ disease patients.

In our research, we observed that serum ANGPTL8 concentrations were positively correlated with LDL-C, TC, and APOB, which suggested that ANGPTL8 might also be involved in lipid metabolism. A study performed by Fenzl et al. found that serum ANGPTL8 is significantly associated with TC, LDL-C, and APOB in patients with long-duration T2DM [25]. In a study of 559 subjects aged 14–28 years, Fu et al. observed that participants in the highest quartile of ANGPTL8 levels had the highest levels of TC, triglycerides, and LDL-C [26]. Sequence variants of the ANGPTL8 gene have been correlated with decreased HDL and LDL levels in Hispanic and African Americans [5]. This observation is in line with the positive correlation between ANGPTL8 and LDL-C in Graves’ disease participants reported here. Another study has shown that ANGPTL8 is a stress-response protein that regulates fat metabolism by suppressing adipose triglyceride lipase (ATGL) expression, revealing a mechanistic connection between ANGPTL8 and lipid homeostasis in mammalian cells [27]. It has been widely accepted that thyroid hormone has major effects on the metabolism of lipids [28–30]. Elevated levels of thyroid hormone in hyperthyroidism are associated with increased clearance of lipolysis and cholesterol. Investigations need to be carried out into whether the low levels of LDL-C, TC, and APOB in Graves’ disease might be mediated by decreased ANGPTL8 release. Given that ANGPTL8 is attracting ever more attention among researchers in the field of metabolic diseases, we recommend that further studies be conducted to elucidate the role of ANGPTL8 in the development of Graves’ disease.

The limitations of our study also require comment. Firstly, this study is limited by its cross-sectional design; thus, only associations (not causation) between serum ANGPTL8 and thyroid function could be addressed. Secondly, we divided subjects into groups according to thyroid function prior to random selection, which may have caused selection bias. Thirdly, serum ANGPTL8 levels may be affected by possible and variable acute stress experienced by the individual subjects. Due to these limitations, more large-scale population-based prospective studies are warranted.

In summary, our results indicate that circulating ANGPTL8 concentrations were significantly decreased in Graves’ disease patients. Moreover, serum ANGPTL8 was negatively correlated with FT3, FT4, and TPOAb, but positively correlated with TSH, TC, LDL-C, and APOB. ANGPTL8 may hence play a role in the pathogenesis of Graves’ disease.
